# Accessible Synthetic Probes for Staining Actin inside Platelets and Megakaryocytes by Employing Lifeact Peptide

**DOI:** 10.1002/cbic.201500120

**Published:** 2015-06-15

**Authors:** Lucia Cardo, Steve G Thomas, Alexandra Mazharian, Zoe Pikramenou, Joshua Z Rappoport, Michael J Hannon, Stephen P Watson

**Affiliations:** [a] School of Chemistry, University of Birmingham Edgbaston, Birmingham B15 2TT (UK) E-mail: m.j.hannon@bham.ac.uk; [b] Centre for Cardiovascular Sciences, College of Medical and Dental Sciences, University of Birmingham Edgbaston, Birmingham B15 2TT (UK); [c] School of Bioscience, University of Birmingham Edgbaston, Birmingham B15 2TT (UK)

**Keywords:** actin, cell-penetrating hybrids, fluorescent probes, FRET, Lifeact

## Abstract

Lifeact is a 17-residue peptide that can be employed in cell microscopy as a probe for F-actin when fused to fluorescent proteins, but therefore is not suitable for all cell types. We have conjugated fluorescently labelled Lifeact to three different cell-penetrating systems (a myristoylated carrier (myr), the pH low insertion peptide (pHLIP) and the cationic peptide TAT) as a strategy to deliver Lifeact into cells and developed new tools for actin staining with improved synthetic accessibility and low toxicity, focusing on their suitability in platelets and megakaryocytes. Using confocal microscopy, we characterised the cell distribution of the new hybrids in fixed cells, and found that both myr– and pHLIP–Lifeact conjugates provide efficient actin staining upon cleavage of Lifeact from the carriers, without affecting cell spreading. This new approach could facilitate the design of new tools for actin visualisation.

## Introduction

Actin is one of the most abundant proteins in eukaryotes that can exist as filamentous actin (F-actin) formed from ATP-mediated polymerisation of monomeric actin (G-actin) and existing in highly dynamic supramolecular organisations. It is one of the major components of the cytoskeleton playing a key role in cell morphogenesis, division and motility; it is regulated and organised by several actin-binding and signalling proteins.[1] Methods to visualise actin dynamics without interfering with their complex activity are particularly important for cell biologists, and one major challenge is to achieve this by using live cell microscopy.[2]

Current approaches to observe real-time F-actin dynamics use the incorporation of fluorescently labelled G-actin during F-actin polymerisation or employ labelled F-actin binding domains (ABDs, usually deriving from actin binding proteins) as fluorescent markers.[3] Among these, a 17-residue peptide sequence, named Lifeact, from the yeast actin crosslinker Abp140 has been identified as the shortest sequence to interact with F-actin.[4] Lifeact is ideally suited as a probe as it has low toxicity and interference with natural actin dynamics as well as the ability to tag a large distribution of actin structures; therefore there is great interest in Lifeact-based markers for imaging actin. However, existing strategies employing Lifeact, as well as other ABDs or labelled G-actin, require fusion with fluorescent proteins (GFP, RFP, etc.) and insertion in cells with genetic modifications or by microinjection techniques;[4b, 5] these procedures do not always guarantee a proper control of the level of the probe in cells, thus affecting its efficiency, reproducibility and toxicity;[6] more importantly, they are not suitable for all cell types or accessible for all research laboratories. Platelets are one significant example of primary cells that cannot be transformed (they are anucleate) or efficiently microinjected (due to their small size, 1–3 μm diameter), therefore real-time actin dynamic studies are limited to platelets isolated from transgenic mice expressing GFP–actin markers.[7]

Cell staining upon incubation with synthetic actin markers, such as labelled phallotoxins or jasplakinolide and their derivatives, are the most employed alternative tools for visualising actin structures by fluorescent microscopy. Bright, high-resolutions images can be achieved thanks to the wide range of fluorophore–phallotoxin conjugates commercially available, but these compounds are either toxic (in fact they have also been investigated as potential cytotoxic drugs), cell impermeable or interfere with actin polymerisation. Consequently their use is typically limited to the study of fixed cells or only for specific experiments in which known effects on actin polymerisation can be taken into account.[8] Attempts to reduce the toxicity of known synthetic actin-binding compounds by designing new derivatives have often been limited by the complexity and costs of syntheses; however, one fluorogenic and cell-permeable actin marker with remarkably reduced toxicity has recently been identified among several synthetic jasplakinolide derivatives.[9] This is a promising tool, although its potential needs to be characterised further as it shows different behaviours in different cell lines.

We aimed to develop alternative synthetic actin markers with improved synthetic accessibility and reduced toxicity; these being the main limits of the few existing compounds. We focus on the markers' suitability for actin staining in human platelets, as developing new approaches for these cells would help to understand important events in thrombosis and haemostasis; we also verify their versatility in megakaryocytes (MKs), which are responsible for platelet production. Due to the known actin affinity/low toxicity combination, fluorescently labelled Lifeact should guarantee minimum interference with actin dynamics (compared to other existing actin binders), therefore it is an ideal component for an actin marker. Because it is not cell permeable, we explore three different carriers able to promote the delivery and release of labelled Lifeact into the cytosol of platelets and MKs. To the best of our knowledge, there is only one successful example of a membrane-permeable synthetic carrier–Lifeact conjugate, which was designed exclusively for live imaging in plant cells, in which Lifeact was fused to the antimicrobial peptide BP100 as a specific vector for these cells.[10]

We investigate three different systems as potential vectors for Lifeact: a myristoylated (myr) carrier,[11] the pH low insertion peptide (pHLIP)[12] and the cationic cell-penetrating peptide (CPP) TAT.[13] We conjugated Lifeact to each of these carriers both through disulfide-based linkers and covalent bonds, and investigated the efficiency of the new hybrids as actin probes and the cell distribution of the carriers by fluorescent confocal microscopy with actin staining as an unambiguous read out.

## Results and Discussion

### Design and synthesis

Platelets are small cells (1–3 μm) that can be readily isolated from blood and kept in buffer for up to 6–8 h. In this study, we investigated three potential carriers for Lifeact, selecting relatively small ones among the large variety of cell-penetrating materials available and known to promote fast cell uptake of their cargos (<30–60 min).[14] Also, they are hypothesised to penetrate cell membranes by different mechanisms, thus we were able to investigate the compatibility of different delivery methods into platelets. Lipid-based carriers, such as the myr system that we employed here, are believed to be independent from membrane-recognition events and are, therefore, more versatile than peptide-based carriers.[11, 15] Furthermore, both palmitoylation and myristoylation have been employed to deliver potential antiplatelet drugs, thus proving their compatibility with platelets.[16] pHLIP is a 38-residue transmembrane peptide helix isolated from bacteriorhodopsin C that is able to deliver several cargos (conjugated at the C terminus by a cleavable link) in response to pH changes: at low pH (between 6.5 and 7.0), pHLIP folds into an α-helix conformation able to penetrate the cell membrane, translocate the cargo and release it upon cytosolic disulfide reduction. An increase in pH (between 7.0 and 7.5) unfolds the α-helix and releases the carrier to the extracellular environment.[17] This carrier is also compatible with platelets; we have recently described its ability to deliver nanomaterials into these cells.[18] Finally, we employed the TAT peptide, which is one of the most common CPPs and widely employed in drug-delivery research.

**Scheme 1:**
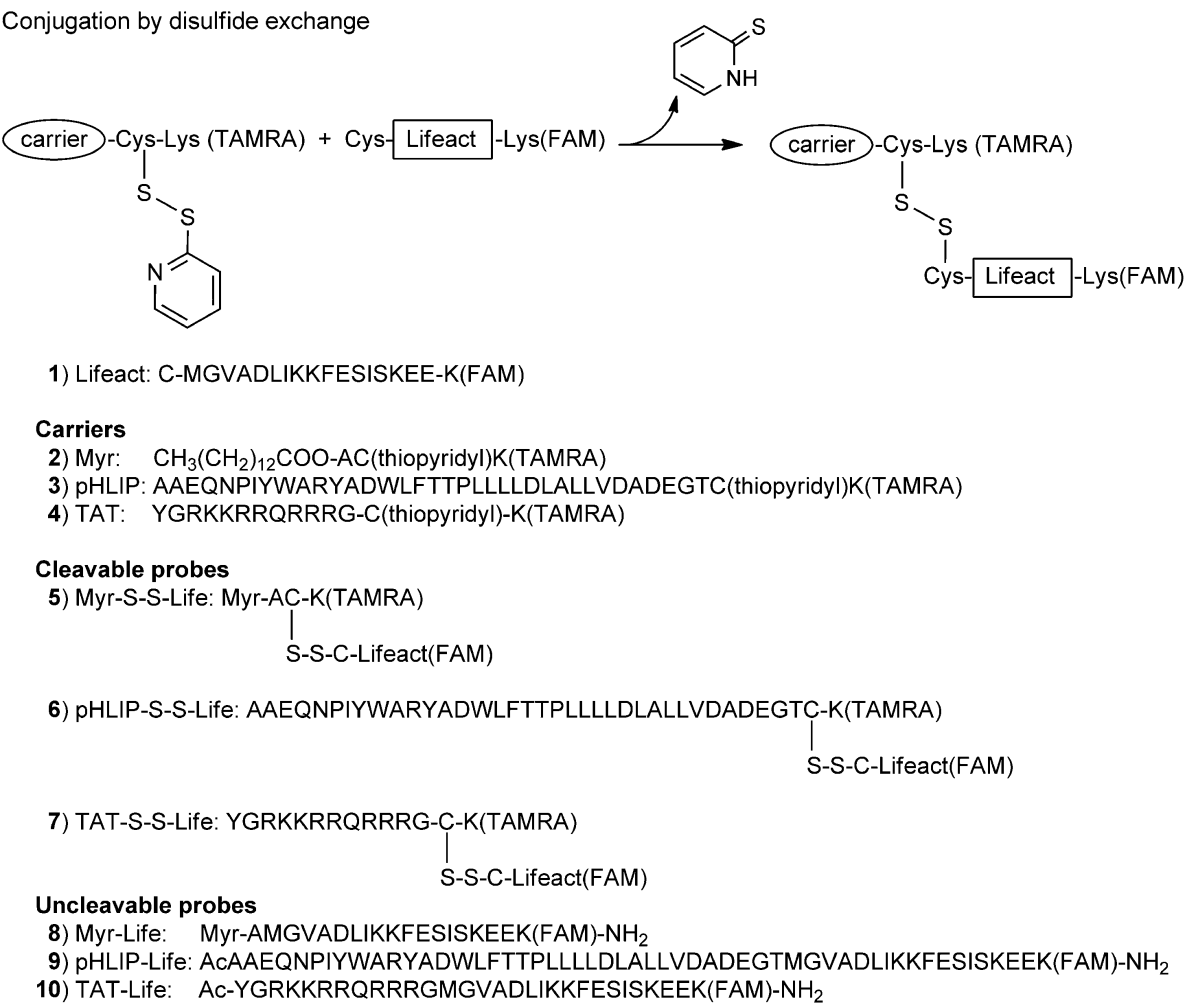
Synthesis of carrier–Lifeact hybrids. Scheme of conjugation between carriers (labelled with TAMRA) and Lifeact (labelled with FAM) by disulfide bond exchange and a list of the cleavable (by disulfide reduction) and uncleavable compounds investigated for cell imaging.

We conjugated Lifeact to each carrier through a disulfide-bond-based linker to allow cleavage and release of the probe in the reductive environment in the cytosol after membrane penetration. We modified Lifeact at the C-terminal by adding a Lys residue labelled with carboxyfluorescein (FAM) so as to allow fluorescent detection of actin staining; at the N-terminal we added a Cys residue for disulfide bond formation with the carrier. Each of the three selected carriers was modified at the C-terminal by adding a Cys followed by a Lys residue labelled with carboxytetramethylrhodamine (TAMRA) for fluorescent detection of the carrier; the thiol group of the Cys was protected by a 2-thiopyridyl group to allow thiol–disulfide exchange with the cargo Lifeact (Scheme 1). We prepared three cleavable compounds: Myr-S-S-Life (**5**), pHLIP-S-S-Life (**6**) and TAT-S-S-Life (**7**). To understand the role of cleavage in Lifeact delivery, we also prepared three analogous uncleavable systems in which carrier and Lifeact are covalently bound: Myr–Life (**8**), pHLIP–Life (**9**) and TAT–Life (**10**).

The compounds were obtained by one-step conjugation between commercial peptides or by following standard and reliable synthetic procedures (peptide synthesis, amino group labelling and disulfide exchange); this is one advantage compared to other actin markers obtained by complex multistep organic syntheses.

The FAM/TAMRA pair (donor and acceptor, respectively) is suitable for FRET spectroscopy and this plays a dual function: providing an additional tool to monitor cleavage and cell uptake[19] and reducing the background fluorescence originating from extracellular or uncleaved Lifeact(FAM). Emission scans of the cleavable compounds **5**, **6** and **7** in the presence of a reducing agent confirm that energy transfer occurs when the disulfide bond is intact and that its cleavage causes a decrease in FRET and a large increase in donor emission intensity (Figure S1).

### Confocal microscopy in fixed platelets and megakaryocytes

**Figure 1:**
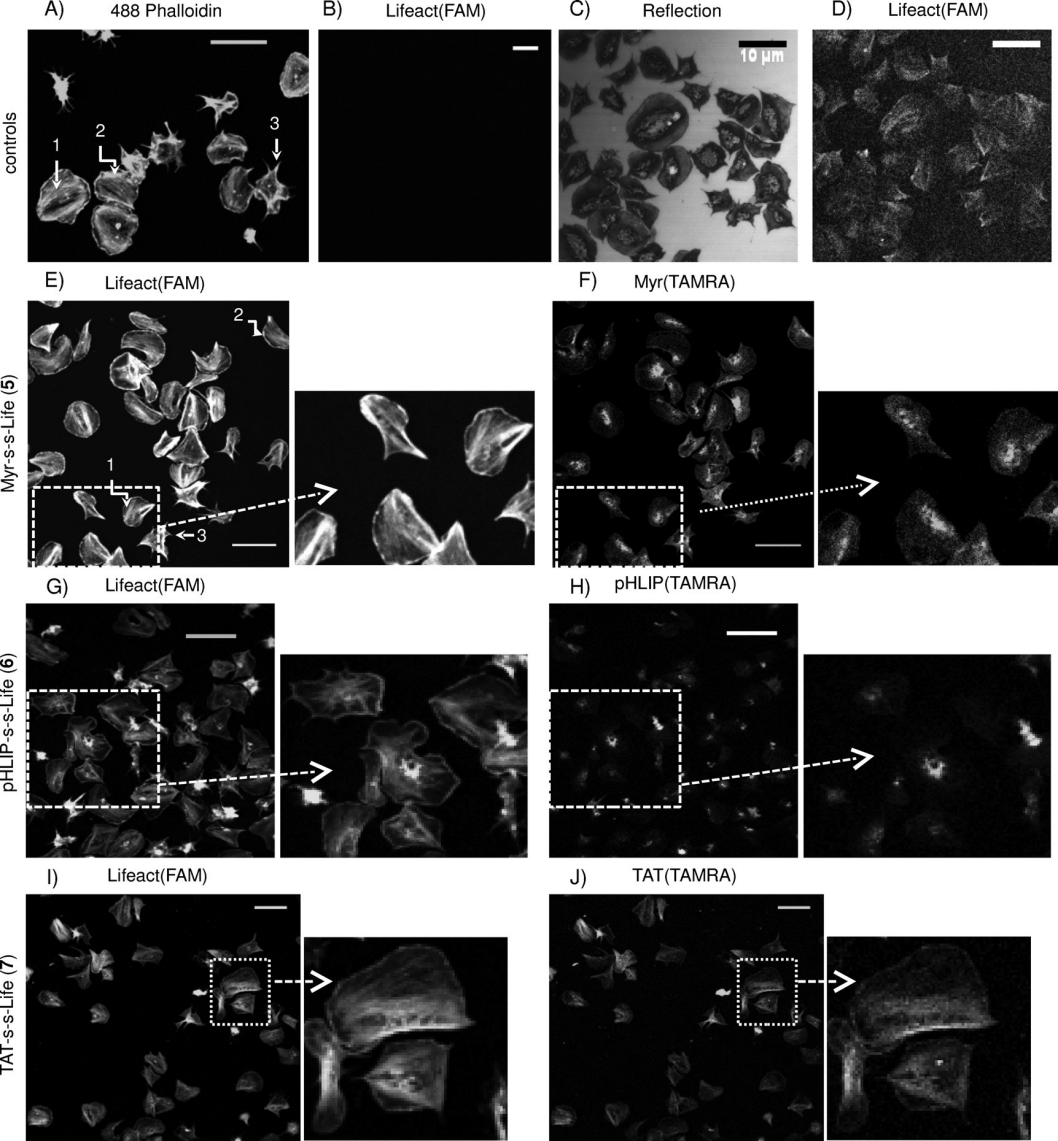
Actin staining in human platelets. Washed platelets were treated with different probes and analysed by confocal fluorescence microscopy. A) Representative actin staining of platelets with Alexa 488 Phalloidin upon fixation and permeabilisation (*λ*_ex_=488, stress 1: fibres, 2: lamellipodia and 3: filopodia). B)–D) Cells treated with Lifeact(FAM) (1) only: B) and D) are fluorescence images at standard gain and upon gain increase, respectively; C) is the corresponding reflection image showing correct platelets spreading. E), G) and I) Lifeact(FAM) detection (actin staining) in platelets preincubated with 5 (4 μm), 6 (4 μm) and 7 (0.5 μm), respectively, spread on fibrinogen and fixed. F), H) and J) Corresponding carrier(TAMRA) detection (*λ*_ex_=543). Representative enlargements are indicated with arrows; scale bars: 10 μm. See also Figures S2 and S3.

We investigated the synthesised compounds' efficiency as actin probes by confocal microscopy in fixed human platelets, aiming to achieve a level of actin staining that is comparable with that observed for Alexa Fluor 488 Phalloidin. Figure 1 A shows phalloidin marking distinctive actin organisations in platelets, such as stress fibres, lamellipodia and filopodia.

Suspensions of washed human platelets (2×10^7^ cells mL^−1^) in Tyrode's buffer were incubated with different carrier–Lifeact systems (concentrations of incubation between 0.5 and 10 μm were investigated), transferred onto cover slips for spreading on fibrinogen and fixed. Detection of FAM and TAMRA emissions by confocal microscopy allowed visualisation of actin staining by Lifeact and the distribution of the carriers in cells, respectively. The image in Figure 1 B confirms that Lifeact(FAM) (**1**) alone does not penetrate cells without an appropriate carrier, as no actin staining or any other fluorescence was observed, although platelets spreading was unaffected (Figure 1 C). Only a large increase in both gain and laser power allowed visualisation of weak fluorescence that could not be recognised as actin filament staining (Figure 1 D). Figure 1 E–L shows fluorescence images of platelets treated with **5** (4 μm, E and F), **6** (4 μm, G and H) and **7** (0.5 μm, I and J). The emission of Lifeact(FAM) and carrier(TAMRA) are on the left and right, respectively, and representative enlargements are indicated. By employing both Myr-S-S-Life and pHLIP-S-S-Life (Figure 1 E and G), we observed normal spreading of platelets and typical staining of common F-actin structures (stress fibres, lamellipodia and filopodia); this is comparable to the staining achieved with phalloidin (Figure 1 A). In both cases, carriers(TAMRA) (Figure 1 F and H) are present inside cells, but clearly separate from Lifeact(FAM) and not involved in actin staining, thus indicating that cleavage between carriers and Lifeact had occurred (additional images in Figures S2 and S3). In contrast, TAT-S-S-Life significantly affects platelet viability; even at relatively low concentrations (1–2 μm) of compound, more than 50 % of cells are incorrectly spread on fibrinogen (Figure S2 O–R). At lower concentrations (0.5–1 μm, necessary to observe emission), platelets treated with this probe present a reduced mean surface area compared to controls and to cells incubated with the other two probes. In addition, both cleavage and actin staining are uncertain as Lifeact(FAM) (Figure 1 I) and TAT(TAMRA) staining (Figures 1 J and S2) appear to overlap.

The procedure employed for sample preparation is based on preincubation of cells with the probes, thereby proving that 1) the new compounds do not need a permeabilisation step, which is necessary for penetration of phalloidin, and 2) cell spreading is not influenced by preincubation with the new systems. However, as a further control, we prepared slides according to the same procedure employed for platelets stained with phalloidin, in which spread cells were fixed, permeabilised and treated with the carrier–Lifeact systems; no significant improvement in actin staining was observed (Figure S4). A range of incubation times (0–45 min) was also explored; this showed that images of platelets preincubated with the probe for 45 min prior spreading are brighter; however, satisfactory staining was also observed when using incubation times of a few minutes (Figure S5), thus indicating fast uptake. We decided to keep incubation times of 30 min for all our experiments.

**Figure 2:**
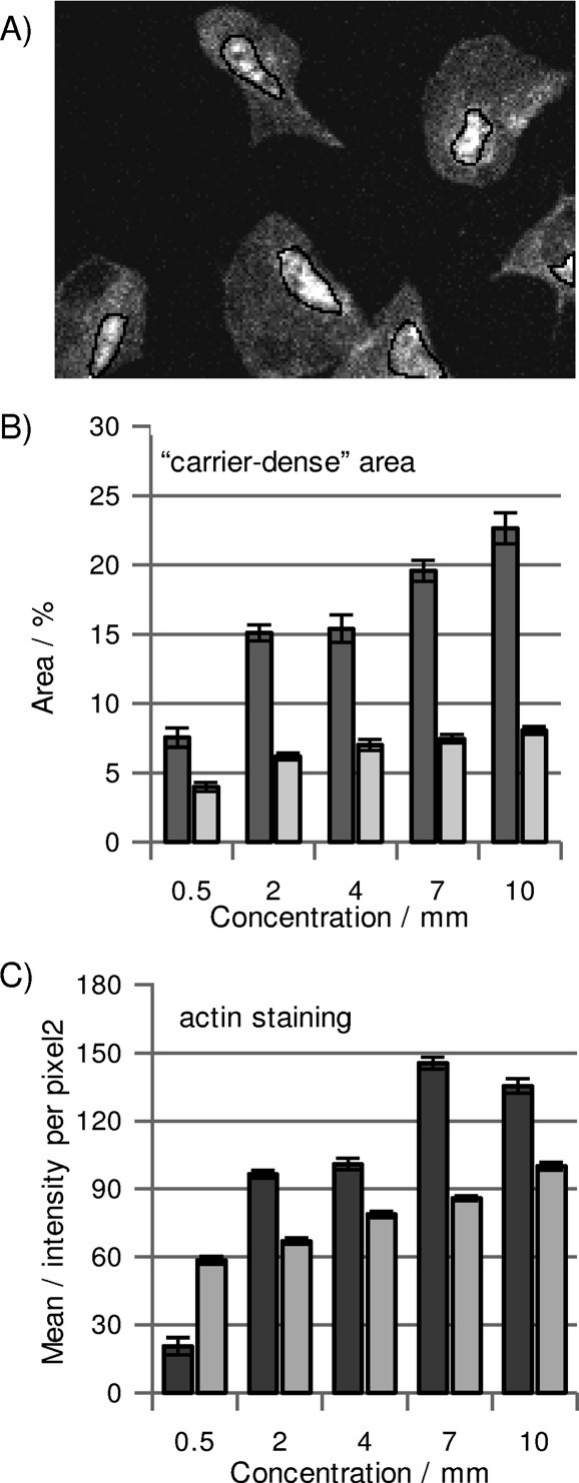
Analysis of probes. Platelets were treated with different concentrations of 5 (▪) and 6 (▪) spread on fibrinogen, fixed and imaged by confocal microscopy. Carrier-dense regions were selected in TAMRA emission images (indicated with black lines in (A)); their mean area [% relative to the total cell area±SEM] was measured in (B). From FAM emission images of the same cells, the mean emission intensities (±SEM) of Lifeact(FAM), which are representative of actin staining, were measured in (C). Each bar is the average of at least 100 measured [area]/[mean intensity] from two different experiments. See also Figures S6 and S7.

In order to characterise and compare Myr-S-S-Life and pHLIP-S-S-Life, we quantified both florescence intensities and areas of relevant regions of the cells. In particular, we observed that both myr and pHLIP carriers accumulated in very distinct regions of the cells, possibly in membrane-dense regions. We selected and analysed these “carrier-dense” regions (black lines in Figure 2 A) using as threshold a ratio between mean TAMRA intensities of carrier-dense areas and mean TAMRA intensities of the corresponding entire cell above 1.5 (for each selected cell). The chart in Figure 2 B reports the percentage area of selected carrier-dense regions relative to the cell area at different concentrations of Myr-S-S-Life and pHLIP-S-S-Life. Generally, “myr-dense” areas are 1.9 times larger than “pHLIP-dense” areas at the lowest concentration of probe and 2.8 times at the highest concentration, thus indicating that myristoylation introduces a higher concentration of carrier stack in cells. Furthermore, the concentration of compound during incubation affects myr-dense areas (threefold increase over the concentration range of 0.5 to 10 μm) more than pHLIP-dense areas (twofold increase over the same range). The higher accumulation of myr in platelets could be simply the consequence of a higher cell uptake of myr–Lifeact hybrid. Alternatively, the different cell distribution observed is consistent with proposed theories about the internalisation processes of these carriers. Myristoylated carriers are believed to penetrate cells based on their affinity for lipid cell membranes, and the concentration-dependent accumulation that we observe in platelets suggests that the vector remains anchored at the platelets' membranes. This is also in agreement with studies in which myristoylation and palmitoylation were employed to enhance the activity of anti-platelet drugs targeting receptors at the inner leaflet of transmembrane proteins.[16c, 20]

As a contrast, we incubated platelets with pHLIP-S-S-Life at pH 6.5 to induce carrier penetration and cargo delivery, followed by washes at pH 7.4 (controls confirmed that this does not affect cell spreading), which should promote release of membrane-attached carrier back to the extracellular environment.[12] This would explain why pHLIP accumulation is lower than that of myr. We also observed significant delocalisation between FAM and TAMRA emission signals in myr-dense regions, whereas the two fluorophores partially colocalise in pHLIP-dense regions (compare images in Figure 1 E and F, and G and H; more details are reported in Figures S2, S3 and S6); this suggests that release of the cargo–Lifeact is more efficient when employing myristoylation rather than pHLIP. This observation is important because detection of Lifeact(FAM) bound to the carrier and not involved in actin staining might lead to misinterpretation in actin studies; however, labelling the carrier allowed uncleaved Lifeact to be identified in relatively small regions (between 3 and 8 % of total cell area); this did not influence the mean emission intensity of FAM (Figure S7). Measurements of mean emission intensity of Lifeact(FAM) inside cells (Figure 2 C), associated with actin staining efficiency, indicate that the brightness of the staining improves when the concentrations of both Myr-S-S-Life and pHLIP-S-S-Life are increased, but higher emission intensities with lower concentrations of probe are achieved with Myr-S-S-Life (additional controls in Figure S7).

**Figure 3:**
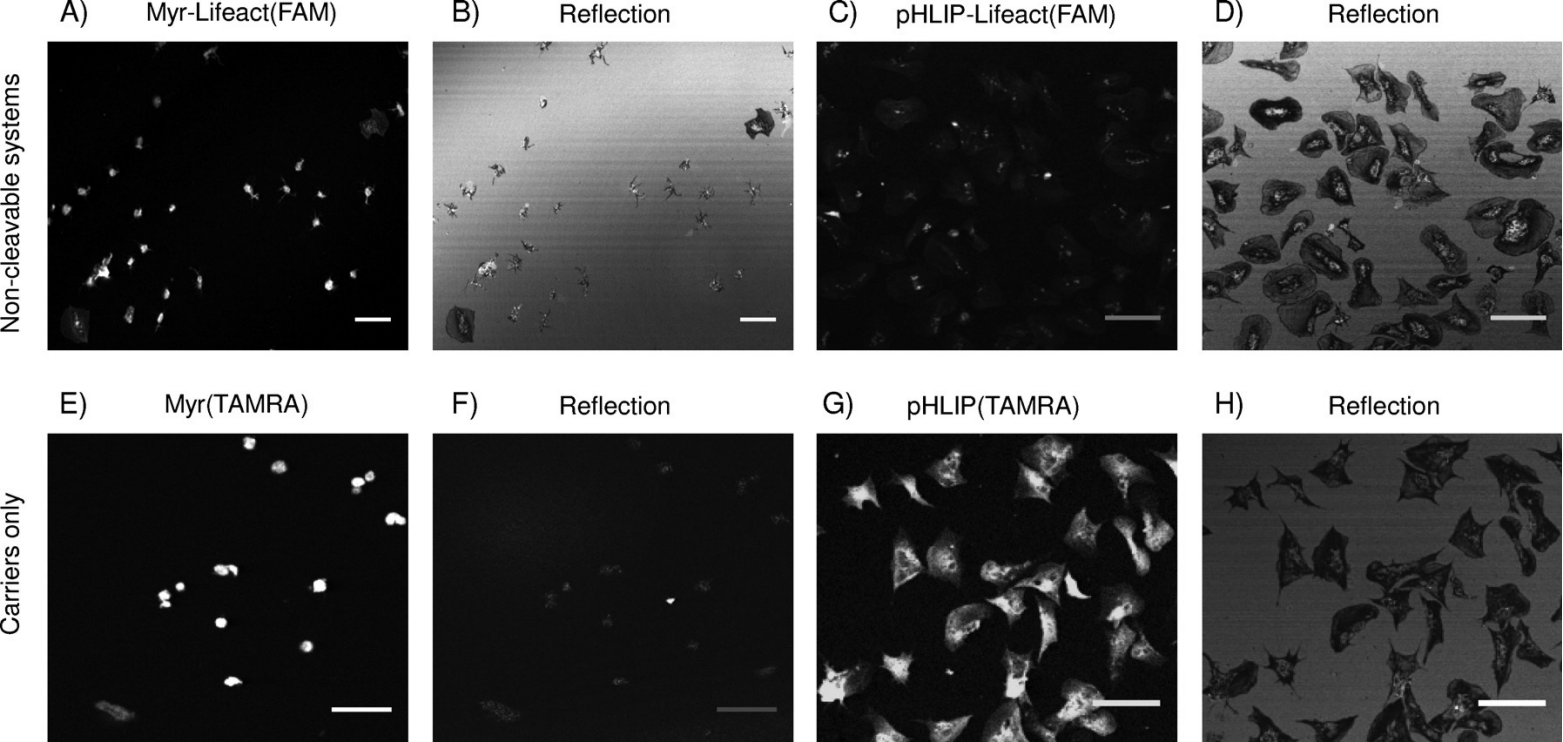
Role of cleavage. Fluorescence images from confocal microscopy of platelets treated with A) uncleavable Myr-Life (8; 1 μm, *λ*_ex_=488 nm), C) pHLIP-Life (9; 4 μm, *λ*_ex_=488 nm), E) carrier Myr(TAMRA) (2; 1 μm, *λ*_ex_=543 nm) and G) carrier pHLIP(TAMRA) (3; 4 μm, *λ*_ex_=543 nm). Corresponding reflection images are shown in (B), (D), (F) and (H). Scale bars: 10 μm.

We treated human platelets with the uncleavable derivatives Myr-Life (**8**; Figure 3 A and B) and pHLIP-Life (**9**; Figure 3 C and D); no actin staining was observed in either case (0.5–10 μm concentrations of compounds were explored), thus indicating that release of Lifeact is essential for its binding with actin. Furthermore, **8** affects platelet spreading even at 1 μm (Figure 3 B). A similar pattern of results was observed when platelets were treated with carriers only: Myr(TAMRA) (**2**; Figure 3 E and F) severely affected cell spreading, although this could be a consequence of carrier insolubility when it is not bound to a peptide, although pHLIP(TAMRA) (**3**; Figure 3 G and H) is randomly distributed in correctly spread cells. Images in Figure 3 A and B reinforce the hypothesis that Myr remains anchored at the membrane in such a way that its covalent link with the actin binder affects cell viability. In contrast, the transmembrane fragment pHLIP is unable to diffuse into the cytosol, as expected, but the covalent binding with Lifeact does not interfere with cell spreading, probably due to a different motility of the system in membranes. We also observed that accumulation of both **9** and **3** (Figure 3 C and G) is broad and random rather than concentrated in relatively small pHLIP-dense regions (as is that of the cleavable pHLIP-S-S-Life); this suggests that the presence of a cleavable cargo influences penetration and/or release of the carrier.

Uncleavable TAT-Life (**10**) and carrier TAT(TAMRA) (**4**) were also tested in platelets. Like the analogous cleavable system, both compounds prevented cell spreading at low concentrations, and staining by TAT-Life had no clear definition (Figure S8), thus confirming the incompatibility of the polycationic TAT with platelets. The failure of a cell-penetrating system could depend on a combination of factors (e.g., the cargo–carrier–fluorophore combination, the type of linker, the type of cell and its set of surface receptors). However, our images show that all TAT-containing compounds affect platelet spreading; this suggests that TAT is the disturbing component. There are a few studies describing how polycationic-containing systems (including polylysine and TAT) influence both platelet aggregation and activation, either by adhering to the negatively charged platelet membranes and forming bridges between adjacent cells or by interfering with specific membrane receptors.[20, 21] These might be related to the behaviour that we observe, and this aspect should be taken into consideration when planning cargo delivery in platelets employing polycationic carriers.

**Figure 4:**
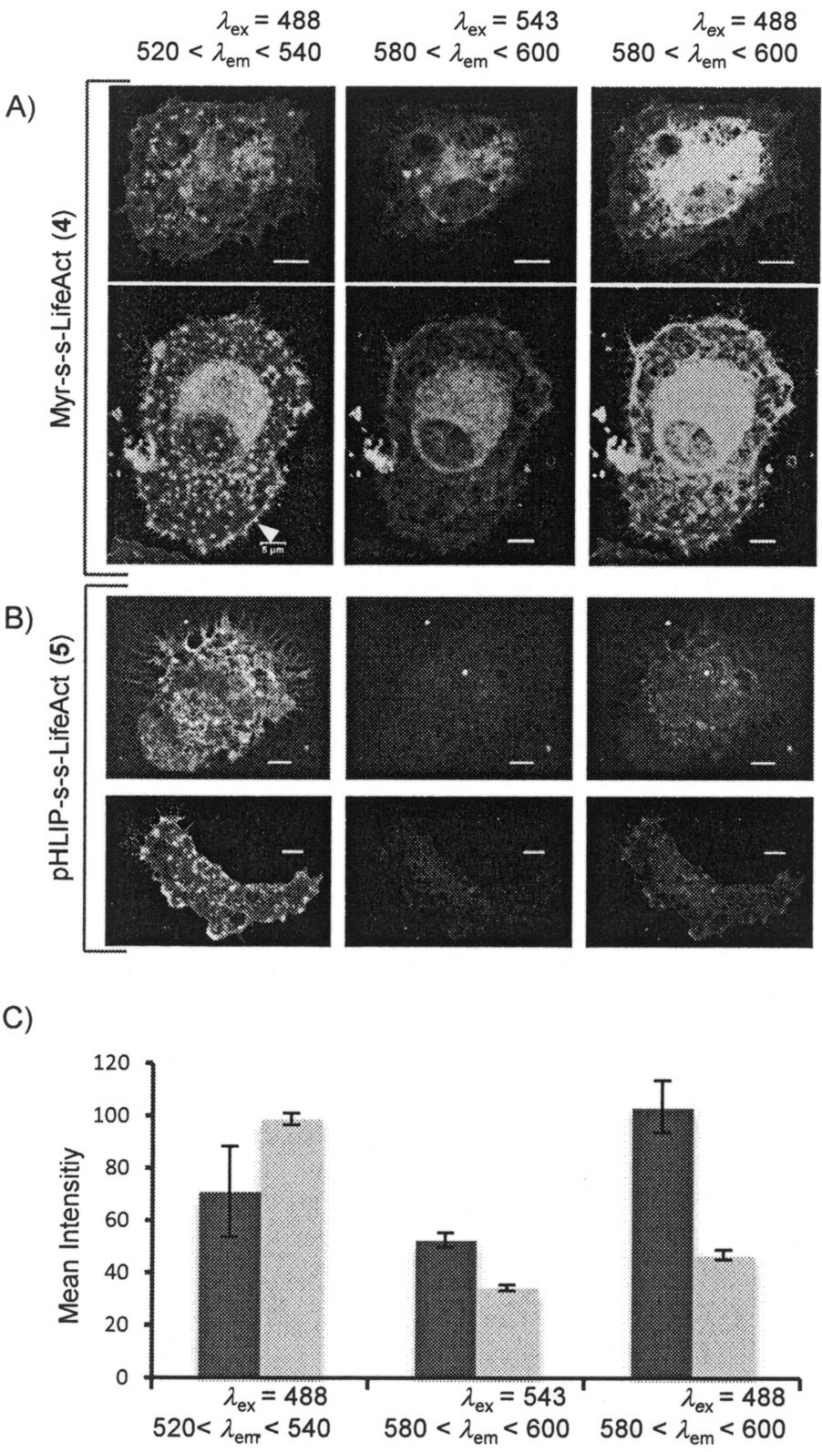
Actin staining in MKs. MKs were incubated with 5 μm of A) 5 or B) 6 spread on fibrinogen, fixed and analysed by confocal fluorescence microscopy. Lifeact(FAM), carrier(TAMRA) and FRET images are on the left, middle and right, respectively. Scale bars: 5 μm. A typical podosome structure is indicated with a white arrow in (A). C) Mean emission intensities (±SEM) of Lifeact(FAM), carrier(TAMRA) and FRET with 5 (▪) and 6 (▪); 20 cells from two different experiments were used for each measurement.

Since **5** and **6** are promising actin markers for platelets, we tested their efficiency in megakaryocytes (MKs), which are responsible for platelet production. Figure 4 A and B shows two representative examples of MKs pretreated with **5** and **6**, respectively, spread on fibrinogen and fixed. Actin staining was observed for compounds (Lifact(FAM) emission images on the left), including marking of typical podosome structures (indicated in the Figure 4 A).[22] As observed in platelets, myristoylated carrier is densely accumulated in cells (carrier(TAMRA) emission images in the middle), whereas the emission of pHLIP is significantly lower. Due to the highest complexity of actin filament organisation in these cells, colocalisation between carriers and Lifeact is complex, and FRET microscopy is important for interpretation: the images on the right were obtained by detecting TAMRA emission caused by energy transfer upon excitation of FAM at 488 nm; this occurs only if Lifeact is anchored to the carrier. FRET intensity was higher than FAM emission intensity in cells treated with **5** (Figure 4 A), thus indicating a large amount of uncleaved compound in cells. Instead, only low FRET was detected in MKs treated with **6** (Figure 4 B), thus indicating low accumulation of uncleaved conjugate (see analysis in Figure 4 C).

In summary, we found that the known cell-penetrating system TAT is not a suitable carrier for platelets, whereas both Myr-S-S-Life (**5**) and pHLIP-S-S-Life (**6**) are efficient probes in both fixed platelets and MKs, displaying F-actin staining properties that are comparable to those of phalloidin. A cytosolic cleavable linker between the carrier and Lifeact is essential to achieve actin staining, and incubation of the cells with the probe (up to 10–20 μm) does not influence their correct spreading on fibrinogen. In the case of myristoylation, cleavage from the cargo is also essential to avoid inhibition of cell spreading. Both myr and pHLIP concentrate in carrier-dense regions in platelets; these are larger when myristoylation is employed (∼15 % of the cell area at 4 μm), although uncleaved Lifeact was not observed. pHLIP-dense regions are significantly reduced compared to myr-dense regions, and a low percentage of uncleaved Lifeact(FAM) was detected, although this did not influence total Lifeact(FAM) emission intensity, related to actin staining, in cells. pHLIP-S-S-Life is also able to deliver and release Lifeact in MKs, whereas Myr-S-S-Life is not suitable for these cells, due to the significant accumulation of uncleaved compound, which would lead to misinterpretation of signals from Lifeact(FAM). Further designs of analogous compounds for MKs should consider inserting the cleavable bond in a more extended linker to facilitate intracellular cleavage.

### Real-time imaging in platelets

**Figure 5:**
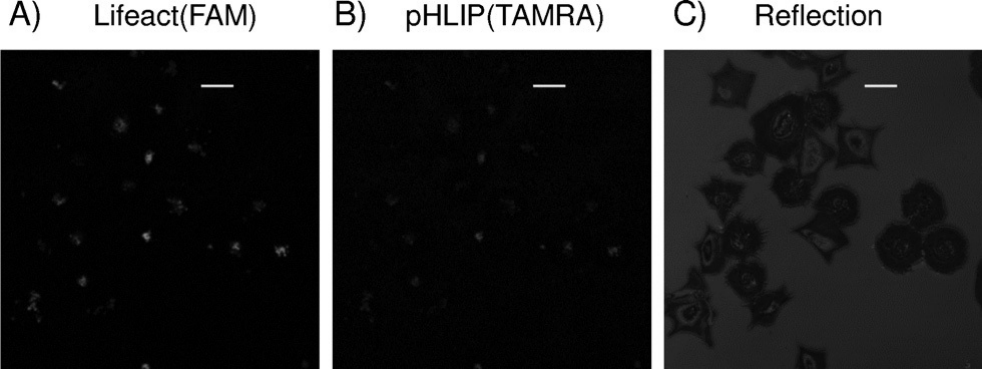
Representative real-time images of human platelets with 6. A) FAM emission, B) TAMRA emission and C) reflection images by confocal microscopy. Scale bars: 10 μm. Platelets spread correctly on fibrinogen (C) but delocalisation between carrier and Lifeact is not evident, thus probe release or Lifeact(FAM) affinity with actin is uncertain. An example real-time image of platelets treated with 5 in given in Figure S9.

Achieving real-time images of F-actin, especially in difficult and important targets such as human platelets, is a critical challenge in cell microscopy. We performed live imaging experiments with **5** and **6**, but the results were not satisfactory. Although reflection images display correctly spreading cells and compound was detected in cell areas, delocalisation between carriers and Lifeact was not observed, and actin staining comparable to that observed in mice platelets by Lifeact-GFP[7] was not achieved (Figures 5 and S9).

**Figure 6:**
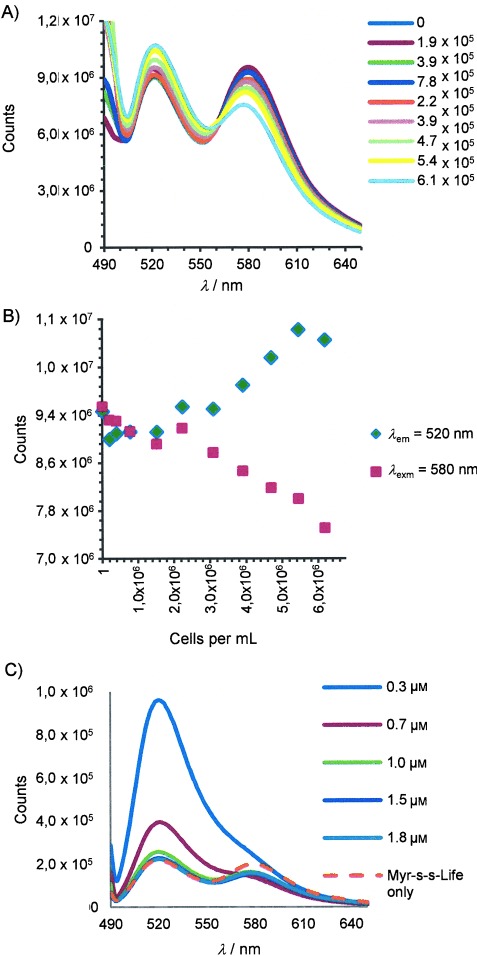
Cleavage of Myr-S-S-Life (5) in the presence of living platelets. A) Emission scans and B) maximum emission intensities of 5 in the presence of increasing concentrations [cells mL^−1^] of human platelets in Tyrode's buffer. C) Emission scans of platelets (2×10^7^ cells mL^−1^) in the presence of increasing concentrations of 5. The scan indicated with a dashed orange line is 0.3 μm 5 without cells.

To verify whether cleavage of compound occurs in the presence of live cells, we measured FRET changes in **5** in the presence of platelets by fluorescence scans (Figure 6). In the control experiment in Tyrode's buffer and in the absence of platelets, upon excitation at 488 nm, the uncleaved compound presents a stable emission profile (monitored for 20 min) with two bands corresponding to FAM (the donor) and TAMRA (the acceptor) at 520 and 580 nm, respectively, as a consequence of energy transfer. Adding increasing concentrations of platelets to a solution of Myr-S-S-Life (1 μm) in Tyrode's buffer, induces an increase in FAM emission and a decrease in TAMRA emission, with the anticorrelation that is typical for FRET decrease (Figure 6 A and B). In a parallel experiment (Figure 6 C), we prepared separate suspensions of human platelets (2×10^7^ cells mL^−1^) in Tyrode's buffer with different concentrations of Myr-S-S-Life: at lower concentration of probe, we only observed FAM emission, thus indicating that most of the compound in solution is cleaved and confirming that platelets cleave the disulfide bond. At the highest concentration, we observed both FAM and TAMRA emission; this must indicate extracellular excess of compound. These experiments demonstrate rapid cleavage of compound in the presence of living platelets (changes in emission were detected immediately after additions) and inside cells, as a reductive environment is necessary for disulfide bond rupture.

Nevertheless, despite the images of fixed cells above and the fact that cleavage occurs as planned in platelets, live cell images remain unsatisfactory. Due to a combination of factors, images of actin in live transgenic mouse platelets labelled with Lifeact-GFP are not nearly as clear as in fixed cell samples:[7] the kinetics of the actin assembling/disassembling process, the reversible nature of the actin–Lifeact interaction, background issues particularly influential in such small cells and the physical-chemical properties of the fluorophore are all key components affecting the quality of images of F-actin in living platelets. Although we do not expect comparable quality between live- and fixed-cell images, these synthetic Lifeact vectors do not give staining that is comparable to Lifeact–GFP, and a few explanations are possible.

The delivery process might not be complete or adequate, and a fixation step might be necessary to promote access to the actin. However, if this were the case, we might expect fixation to have the same beneficial effect on uncleavable Myr-Life or Lifeact only, which it does not. Moreover, the live-cell experiments in Figure 6 clearly indicate that cytosolic release of Lifeact(FAM) occurs and that it is complete when suspensions of platelets (2×10^7^ cells mL^−1^) are treated with concentrations of hybrids <1 μm.

Alternatively the Lifeact–FAM combination might not be the most appropriate marker in terms of affinity for F-actin: the position and/or the nature of the fluorophore and/or the linker between the fluorophore and Lifeact might affect the interaction with F-actin resulting in poor visualisation of dynamic events in real time. For example, Lukinavicius et al. identified one efficient actin marker for live-cell imaging among a library of many derivatives, and modifications to the linker between the fluorophore and the actin binder were key determinants of activity.[9b] An et al. used pHLIP to deliver phalloidin into cancer cells as an antiproliferative drug and observed that the presence of fluorophore on both carrier and cargo strongly influenced the efficiency of the antiproliferative activity.[23] Finally, the plant-cell-specific cell-penetrating carrier–Lifeact hybrid described by Eggenberger et al.[10] highlights the importance of the position of the fluorophore/carrier relative to the Lifeact sequence and suggests that involving the C terminus of Lifeact in the conjugation might affect the binding affinity with actin.

Thus, these myr- and pHLIP–Lifeact conjugates are suitable as scaffolds for further elaboration and optimisation that might enable live- as well as fixed-cell imaging. However, future design aiming to enhance the affinity of the probe for F-actin must be undertaken with caution, as this might also affect the kinetics of actin assembly/disassembly, and the measurement of “biological” real time, which is the ultimate goal.

## Conclusions

There is a great interest in developing new methods to visualise F-actin dynamics by cell microscopy, especially because current methods cannot be applied to all cell types or rely on the use of synthetic markers that are toxic or difficult to modify. We have designed new systems for actin staining with improved synthetic accessibility by employing the 17-residue sequence Lifeact as the actin-binding component so as to ensure low interference with actin filaments and improve toxicity. We conjugated Lifeact to three different cell-penetrating carriers and investigated their ability to promote the delivery and release of Lifeact, and consequently actin staining, in difficult targets such as human platelets and megakaryocytes. We found that the cationic carrier TAT is not suitable for these cells, whereas both a myristoylated carrier and pHLIP, a pH-responsive peptidic vector, promote Lifeact insertion without affecting cell spreading. Actin staining, comparable with that of commercially available phalloidin was observed in fluorescent images of fixed cells upon cytosolic release of the cargo–Lifeact. We highlight the importance of both carrier and cargo labelling, possibly with a pair of fluorophores able to provide a cleavage-responsive signal such as FRET, as tools to achieve a better understanding of our hybrids.

Our studies confirm that bringing together delivery vectors, cleavage sites and labelled Lifeact by employing fast and versatile synthetic procedures, provide a “plug and play” accessible strategy useful for simplifying the design of new actin markers with customisable physical, chemical and biological features. This valuable approach might facilitate the screening and identification of systems suitable for real-time images, bearing in mind that the binding affinity of potential probes with actin filaments, which is necessary for satisfactory visualisation of dynamic events, must not affect their kinetics.

## Experimental Section

Experimental procedures and instruments employed are detailed in the Supporting Information. Here we briefly describe principal methods employed.

**Synthesis**: The following peptides were purchased from Peptide Protein Research Ltd. (Fareham, UK): Lifeact (**1**), pHLIP(TAMRA) (**3**) and TAT(TAMRA) (**4**) were supplied with certified purity >80 % and employed for the following disulfide-exchange steps without further purification; the uncleavable systems Myr-Life (**8**), pHLIP-Life (**9**) and TAT-Life (**10**) were supplied with certified purity >98 % and employed for cell microscopy studies without further purification. Both FAM and TAMRA were conjugated at the α-amino group of the C-terminal lysine residue.

Myr (TAMRA) (**2**) was synthesised by Fmoc solid-phase synthesis followed by Cys protection with thiopyridyl group and Lys labelling with TAMRA. All cleavable compounds Myr-S-S-Life (**5**), pHLIP-S-S-Life (**6**) and TAT-S-S-Life (**7**) were obtained by mixing 1 equiv of carrier **2**, **3** and **4**, respectively, with 2 equiv of **1** in DMF at room temperature for 4 h. All reactions were monitored by analytical RP-HPLC (0–100 % CH_3_CN/0.05 %TFA in H_2_O/0.05 % TFA over 40 min), the compounds were purified by semipreparative RP-HPLC (using the same gradient) and characterised by mass spectrometry. All the peptides were prepared in 150 μm solutions in Tyrode's buffer (134.0 mm NaCl, 2.90 mm KCl, 0.34 mm Na_2_HPO_4_**⋅**12 H_2_O, 12.0 mm NaHCO_3_, 20.0 mm HEPES, 1.0 mm MgCl_2_, pH 7.3). Concentrations were checked by comparing the UV/Vis bands of the dyes, and aliquots were kept at −20 °C until their use for cell biology studies.

**Cell microscopy**. Suspensions of washed platelets (300 μL, 2×10^7^ cells mL^−1^) were preincubated with different volumes of probe solutions (150 μm, 0.5–10 μm concentration of probe during incubation was the range explored) in Tyrode's buffer containing glucose (5 mm, pH 7.4) at 37 °C for 30 min. Suspensions were transferred onto glass coverslips precoated with fibrinogen and allowed to spread at 37 °C for 45 min. For pHLIP–Lifeact systems, incubation and spreading were in Tyrode's buffer containing glucose (5 mm) at pH 6.5. Spread cells were washed with phosphate-buffered saline (PBS), fixed with 10 % formalin, treated with NH_4_Cl_2_ (50 mm), washed again with PBS and mounted on slides for microscopy. The same procedure was employed to stain MKs, except that cell suspensions of 5×10^8^ cells mL^−1^ were employed for incubation and spreading was allowed in medium (and not buffer) for 3 h prior to fixation and mounting. For control platelets stained with phalloidin, platelet suspension (2×10^7^ cells mL^−1^ in Tyrode's buffer) was allowed to spread on glass coverslips precoated with fibrinogen for 45 min at 37 °C, fixed with formalin, permeabilised with 0.1 % Triton X-100 in PBS and stained with Alexa Fluor 488 Phalloidin (15 nm) for 30 min at room temperature. Stained cells were washed with PBS and deionised water and mounted on slides for microscopy.

To allow proper comparison between different compounds, the same microscope parameters (PMT, enlargement, laser power) were employed in all the experiments (unless indicated differently). Samples were imaged by using the 488 (Ar/ArKr laser), 543 and 633 nm (He/Ne laser) laser lines on a Leica DMIRE 2 laser scanning confocal microscope with 63×, 1.4 N/A oil objective. Single images were collected by selecting the best optical plane (i.e., the one that looked most in focus) and using averaging (3 scan accumulations) to improve S/N. Post-imaging analyses (cell selections, mean area and mean fluorescence intensity calculations) were performed by using ImageJ 1.48.

## Supporting Information

As a service to our authors and readers, this journal provides supporting information supplied by the authors. Such materials are peer reviewed and may be re-organized for online delivery, but are not copy-edited or typeset. Technical support issues arising from supporting information (other than missing files) should be addressed to the authors.

miscellaneous_information
