# Supplementary material for: Accessible Synthetic Probes for Staining Actin inside Platelets and Megakaryocytes by Employing Lifeact Peptide
Source: Chembiochem. 2015 Jun 15;16(11):1680–8. doi: 10.1002/cbic.201500120 (PMC4524417; doi:10.1002/cbic.201500120)
Supplement: Supplementary file 1 — miscellaneous_information [file cbic0016-1680-sd1.pdf]

CHEMBIOCHEM

Supporting Information

Accessible Synthetic Probes for Staining Actin inside Platelets and Megakaryocytes by Employing Lifeact Peptide

Lucia Cardo,[a, b] Steve G. Thomas,[b] Alexandra Mazharian,[b] Zoe Pikramenou,[a] Joshua Z. Rappoport,[c] Michael J. Hannon,*[a] and Stephen P. Watson*[b]

cbic_201500120_sm_miscellaneous_information.pdf

Supporting Data

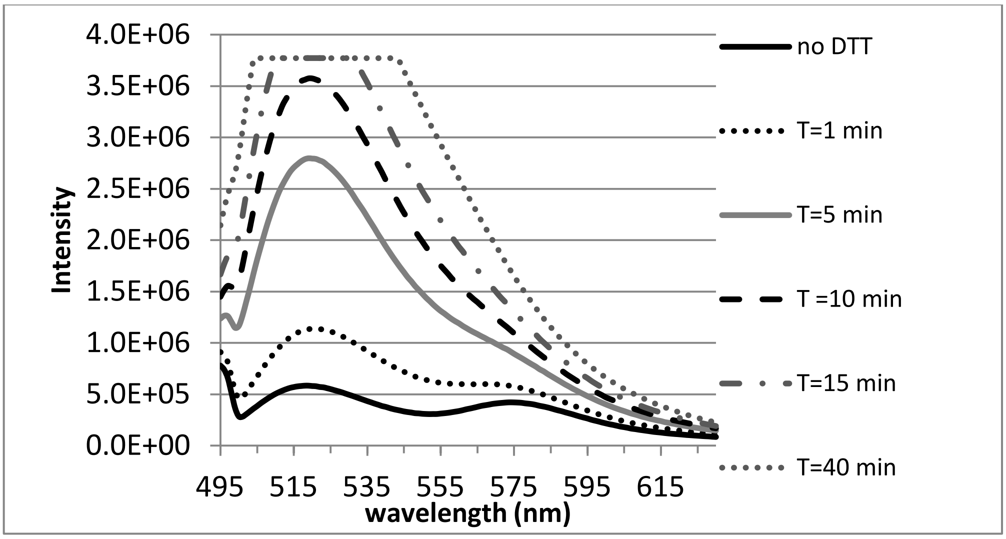

Figure S1. Emission scans (λex=490 nm) of Carrier(TAMRA)-s-s-Lifeact (FAM) (0.5 μM) in the presence of 5 eq. of Dithiothreitol (DTT) in Tyrode's buffer (pH 7.4). The same solution was detected for 40 min (legend indicates the time at which each scan was recorded after the addition of DTT). The solid black line corresponds to the emission of the uncleaved probe before addition of DTT: the emission at 575 nm (TAMRA) with intensity comparable to the emission at 520 nm (FAM) indicates that energy transfer between the two fluorophores occurs. Addition of DTT causes immediate increase of the emission intensity of the donor (FAM) whilst the emission of the acceptor (TAMRA) is not longer detected. In the time of this analysis, saturation of emission signal was observed before emission intensity was stabilized.

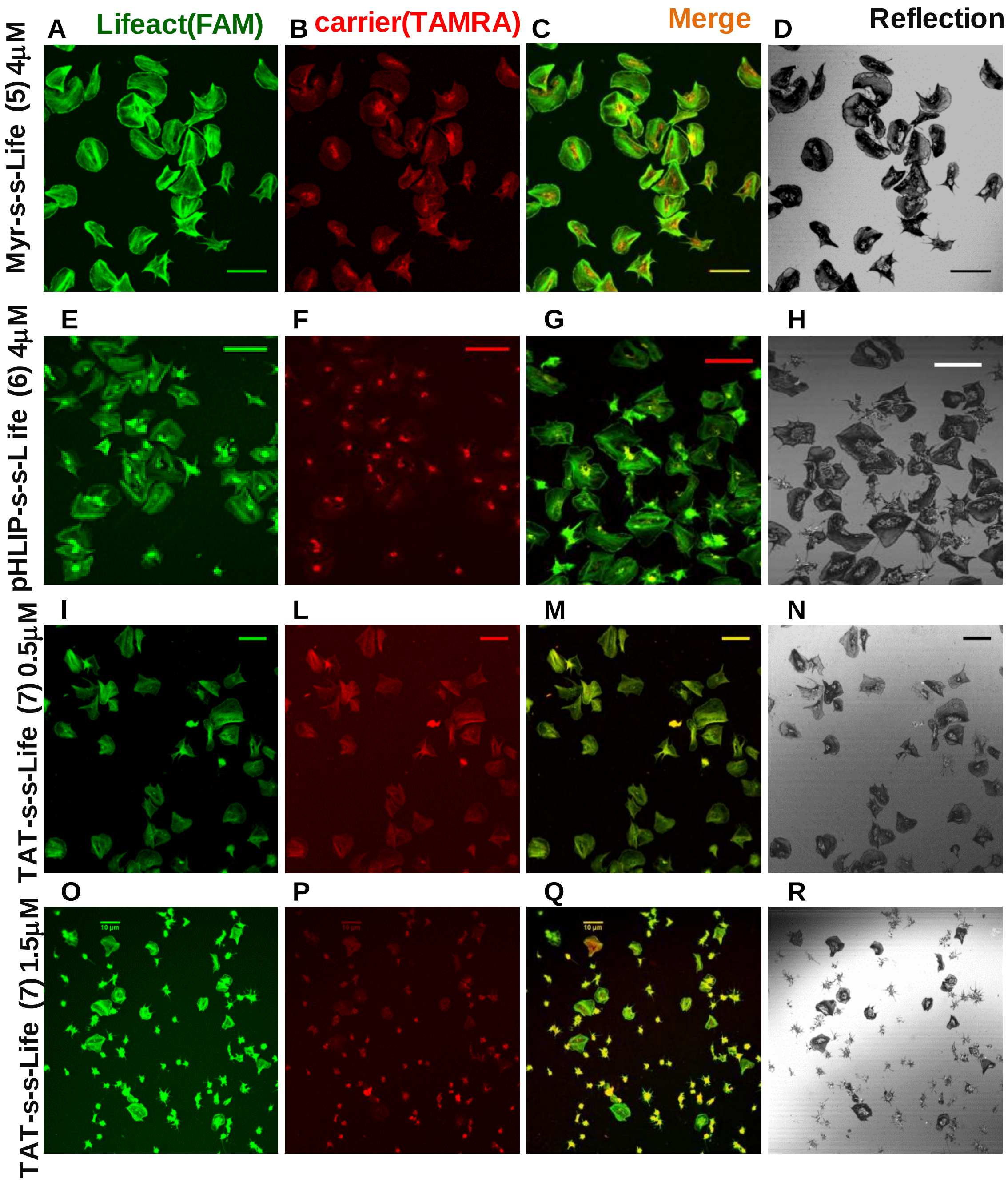

Figure S2. Human Platelets with cleavable probes. Colour images of platelets treated with the 3 cleavable probes, as described in Figure 2. C, G, M and Q shows qualitative co-localization upon merging green and red channels (Lifeact(FAM) and carrier(TAMRA) respectively), whilst D, H, N and R are reflection images showing platelets spreading and confirming complete staining. O to R, where platelets are treated with 1.5 μM of TAT-s-s-Life, show how spreading is affected already at this concentration; I to R show how de-localization between the two colours is not evident, in contrast with the other two systems.

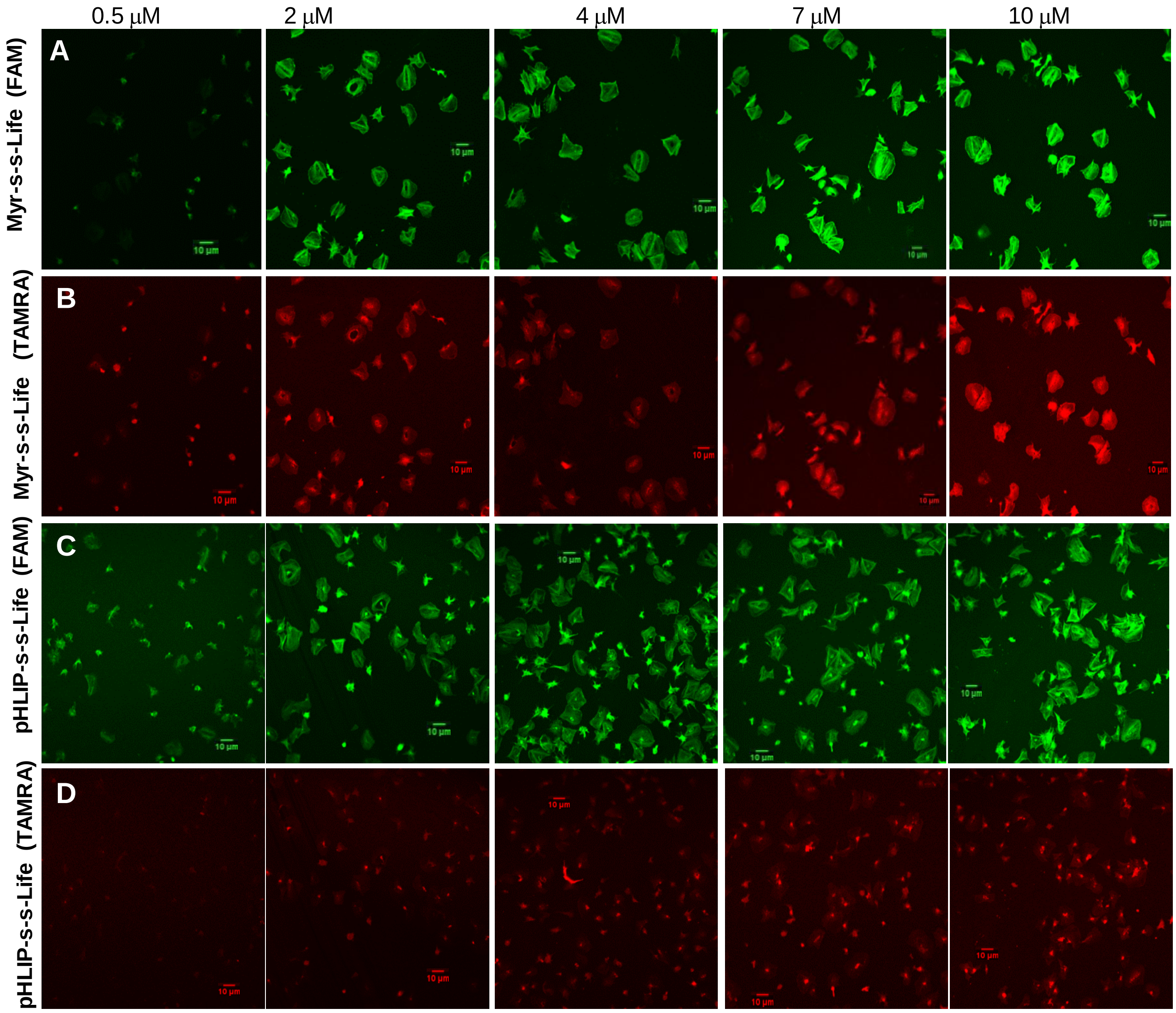

Figure S3. Human Platelets with different concentrations of Myr-s-s-Life (5) (A and B) and pHLIP-s-s-Life (6) (C and D): platelets were incubated with different concentrations (0.5 to 10 μM) of carrier-Lifeact systems, spread on fibrinogen and fixed. Fluorescence images by confocal microscopy were collected to visualize Lifeact (FAM emission using λ_{ex}=488 nm) and carrier (TAMRA emission using λex=543); scale bar 10 μM. Cell spreading and cell size are not affected by probe concentration. Actin staining is more efficient at highest concentrations (A and C). 'Carrier dense' regions are observed using both carriers, although these are larger when myr is employed. 'Myr dense' regions, detected by TAMRA emission, do not present corresponding FAM emission (A and B). Differently, co-localization between 'pHLIP dense' regions, detected by TAMRA emission, and Lifeact that is not involved in actin staining, detected by FAM emission (C and D), is evident suggesting that pHLIP accumulated in those regions are still attached to the cargo or stack in the same region.

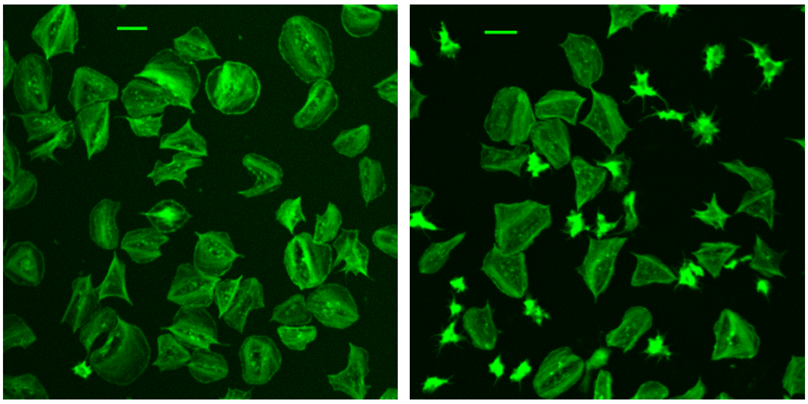

Figure S4. Human Platelets stained with Myr-s-s-Life (5) employing 2 different procedures: in the image on the left, platelets (300 μL, 2x10^7 cell/mL) were incubated with 4 μM of Myr-s-s-Lifeact (30 min, at 37°C), spread on fibrinogen and fixed; image on the right, platelets (300 μL, 2x10^7 cell/mL) were spread on fibrinogen, fixed, permeabilized with 0.1% Triton X-100 in PBS, and treated with 4 μM of Myr-s-s-Life (30 min, at 37°C). Permeabilization does not improve staining (FAM emission using λ_{ex}=488 nm, scale bar 5 μm).

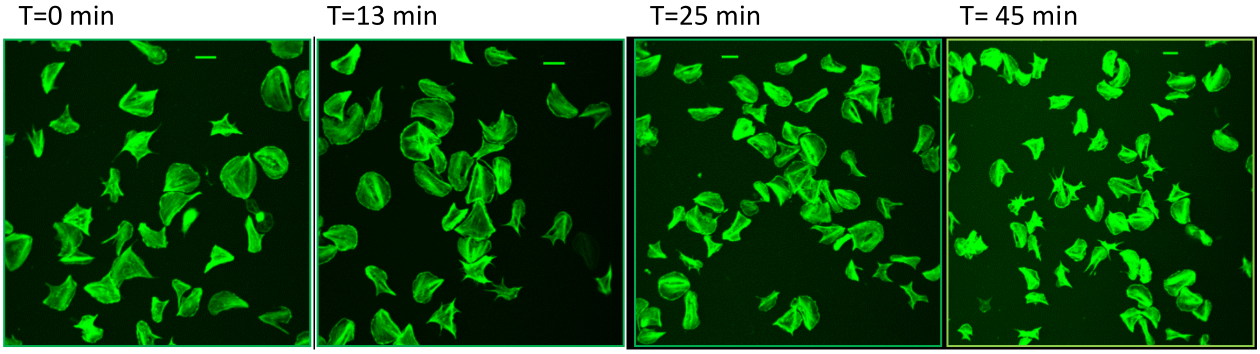

Figure S5. Human Platelets stained with Myr-s-s-Life (5) employing different incubation times: platelets (300 μL, 2x10^7 cell/mL) were incubated with 4 μM of Myr-s-s-Lifeact at 37°C and using different incubation times (0, 13, 25 and 45 min, as indicated in the images); they were transferred on fibrinogen for spreading and fixed; Lifeact(FAM) emission (using λ_{ex}=488 nm) shows that incubation time of 45 min yields brighter images, although actin staining is satisfactory even without incubation but just transferring the platelets/probe mixture directly for spreading (scale bar 5 μm).

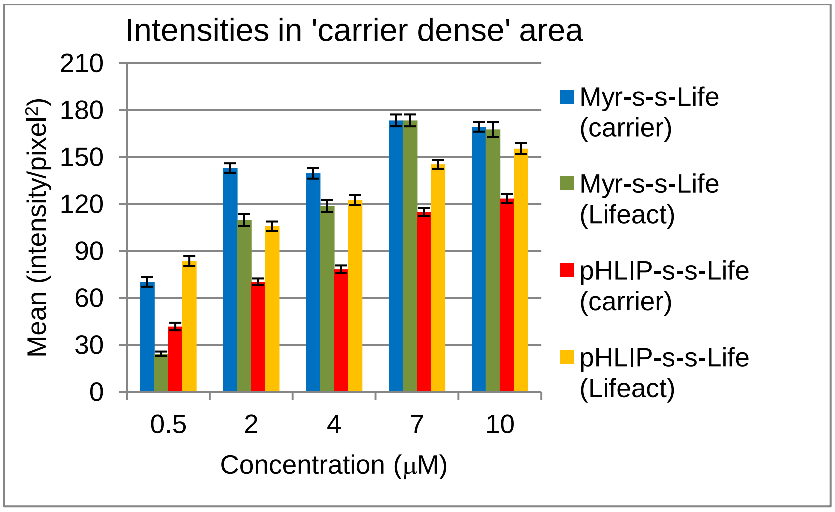

Figure S6. **Intensities in 'Carrier dense' area**. Mean fluorescence intensities of carriers (TAMRA) and Lifeact (FAM) in selected 'carrier dense' areas were measured (±SEM) employing the same regions selected for charts in Figure 3.

TAMRA emission in selected 'myr dense' regions increases by increasing the concentration of Myr-s-s-Life (blue bars, 2.5 folds going from 0.5 to 10 μM). At each concentration, the mean intensity of Lifeact(FAM) emission in the same region is lower or comparable (green bars) and probably deriving only from Lifeact involved in actin staining (also evident from images in Figure 2 and Figure S3). The mean intensity of pHLIP(TAMRA) emission (red bars) in 'pHLIP dense' regions also increases with compound concentration but it is always lower than the corresponding mean intensity of Lifeact(FAM) (yellow bars) where probably the emission of both cleaved actin staining Lifeact and uncleaved Lifeact contribute.

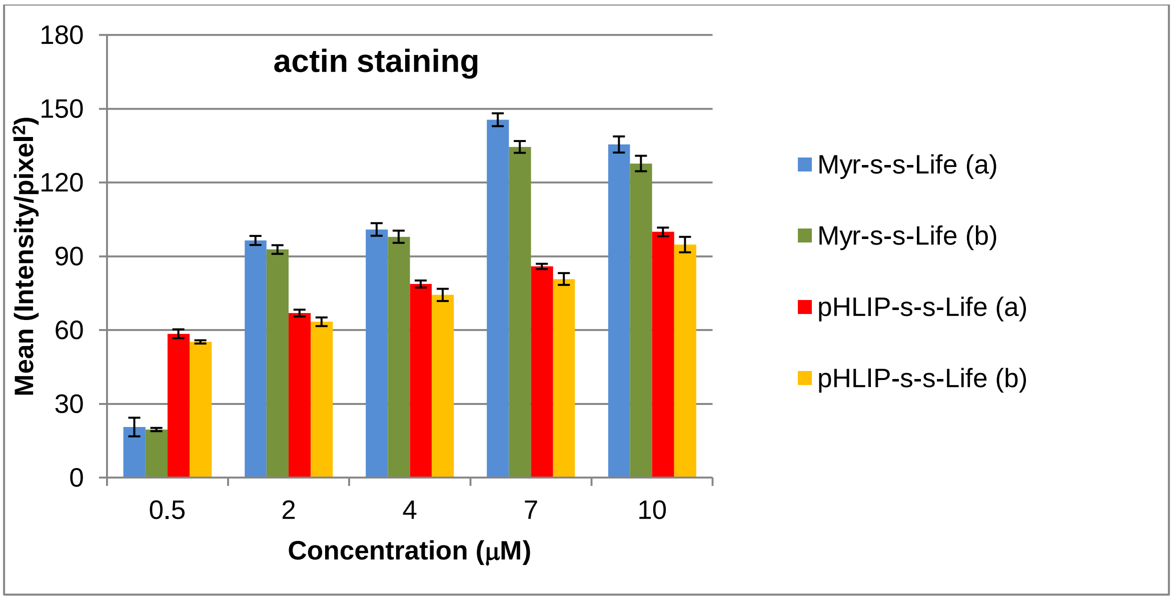

Figure S7. Actin staining. Measurement of Mean emission intensity of Lifeact(FAM) using different concentrations of Myr-s-s-Life and pHLIP-s-s-Life (±SEM). We carried out two types of measurements for each compound: i) we measured the mean intensities of Lifeact(FAM) inside entire cells (blue and red bars, like in Figure 3.B) and ii) the mean intensities of Lifeact(FAM) inside entire cells with subtraction of the mean intensities of Lifeact(FAM) of 'carrier dense' regions (green and yellow bars). The difference between the two types of measurements is constant, indicating that the presence of uncleaved Lifeact, eventually not involved in actin staining, is not influencing the comparison between the two compounds.

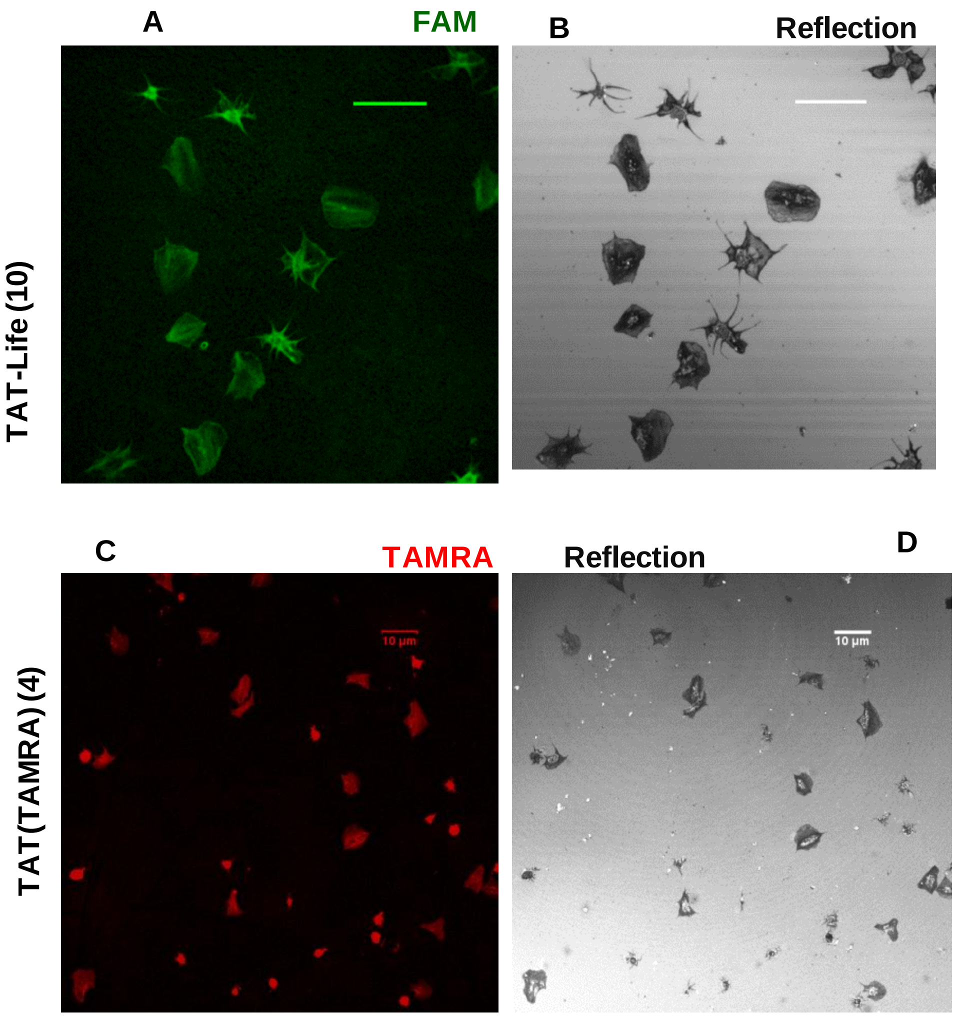

Figure S8. Human Platelets with TAT-Life (10) and (TAT)TAMRA (4). Washed platelets were incubated with different concentrations (0.5 µM in these images) of TAT-Life (10) (A and B) and TAT(TAMRA) (4) (C and D) spread on fibrinogen and fixed. As observed with cleavable TAT-s-s-Life in Figure 2 and S2, concentrations above 0.5-1 µM of TAT-Life inhibit cell spreading and actin staining in spread cells is uncertain. With the same procedure, platelets were treated with TAT(TAMRA) (4) and even less spread cells were observed using 0.5-1 µM of compound (scale bar 10 µm).

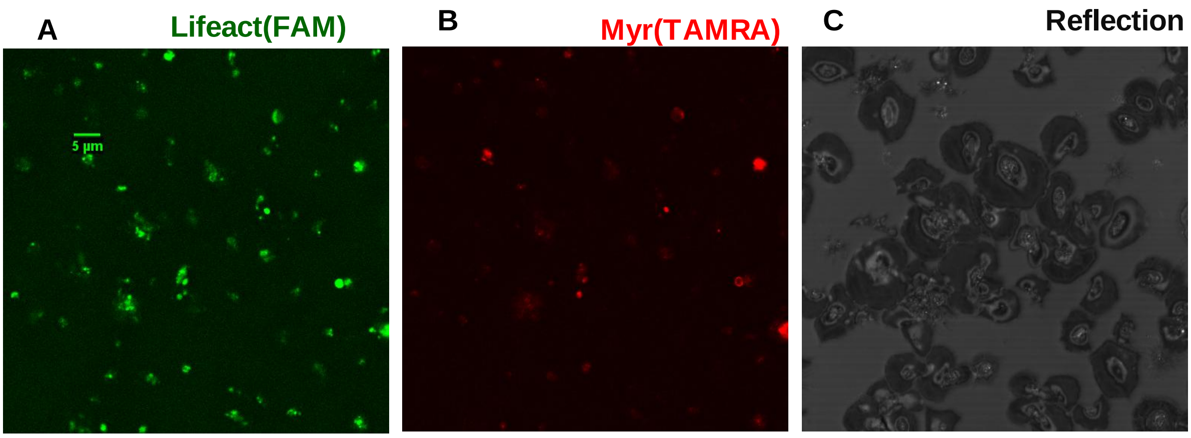

Figure S9. Representative real time images of human platelets with Myr-s-s-Life (5). FAM emission (A), TAMRA emission (B) and reflection (C) images by confocal microscopy (scale bar 5 μm). Platelets spread correctly (C) on fibrinogen but de-colocalization between carrier and Lifeact is not evident, making uncertain probe release or Lifeact(FAM) affinity with actin. Several modifications of experimental procedures, such as varying incubation times, concentrations of probes/cells and wash techniques, were not effective.

Supporting Experimental Procedures

Materials and Instruments

Chemicals where purchased from Sigma-Aldrich with the exception of Rink Amide MBHA resin and Fmoc- protected amino acids for peptide synthesis, purchased from Merck. Solvents for synthesis and HPLC where purchased from Fischer Scientific and dimethylformamide for peptide synthesis was supplied by AGTC bioproducts. Reverse phase high performance liquid chromatography (RP-HPLC) analyses and purifications were performed on Dionex Summit HPLC systems with Chromoleon software, using HPLC grade solvents, supplied by Fisher. Analytical and semipreparative HPLC were acquired with the aid of a Summit P580 quaternary low pressure gradient pump with built in vacuum degasser, while a high pressure gradient pump was employed on the same machine for preparative HPLC. Phenomenex Luna C18 (2) columns were used for analytical (250 x 4.6 mm, 1 ml/min flow), semipreparative (250x10 mm, 3 mL/min flow) and preparative (250 x 21.2 mm, 21 ml/min flow) RP-HPLC, from 0 to 100% Acetonitrile 0.05%TFA in water 0.05% TFA in 40 min was the gradient employed.

Probe concentrations were detected by recording UV-vis scans using a Varian Cary 5000 dual beam spectrometer. Emission scan of the probes in the presence of cells were recorded at 37°C using a Edinburgh Instruments FLS920 spectrophotometer.

Synthesis

All peptides were protected by amidation and acetylation at the C- and N-terminal respectively. The following peptides were purchased from Peptide Protein Research Ltd (UK):

I. **Lifeact (1)**: CMGVADLIKKFESISKEEK(FAM)
II. **pHLIP(TAMRA) (3)**: AAEQNPIYWARYADWLFTTPLLLLDLALLVDADEGTC(thiopyridyl)-K(TAMRA)
III. **TAT(TAMRA) (4)**: YGRKKRRQRRRGC-(thiopyridyl)-K(TAMRA)

IV. **Myr-Life (8)**: Myr-AMGVADLIKKFESISKEEK(FAM)

V. **pHLIP-Life (9)**: AAEQNPIYWARYADWLFTTPLLLLDLALLVDADEGTMGVADLIKKFESI-SKEEK-(FAM)

VI. **TAT-Life (10):** YGRKKRRQRRRGMGVADLIKKFESISKEEK(FAM)

The first three peptides were supplied with certified purity >80% and employed for following disulphide exchange steps without further purifications; the last three uncleavable systems, were supplied with certified purity >98% and employed for cell microscopy studies without further purifications.

$CH_3(CH_2)_{12}$COO-ACK was synthesized by solid phase synthesis based on Fmoc chemistry, using Rink Amide MBHA resin, standard Fmoc protected amino acids and $CH_3(CH_2)_{12}COOH$ (myristic acid) was coupled as last step of synthesis. Deprotection and cleavage from the resin was performed in trifluoroacetic acid (TFA)/water/1,2-Ethanedithiol/ Triisopropylsilane (9.4/0.25/0.25/0.1) for 1 hour at room temperature followed by precipitation with diethyl ether and purification by RP-HPLC. ESI (calc. 531) *m/z* found 531.1 = [M^+]; 553.1=[$M+Na^+$].

$CH_3(CH_2)_{12}$COO-AC(thiopyridyl)K was synthesised by mixing 0.3 eq of ($CH_3(CH_2)_{12}$COO-ACK with 0.6 eq of 2,2′-dithiopyridine in 10 mL of 4% acetic acid in methanol and stirring at RT for 2 hours. The reaction was monitored and the compound purified by RP-HPLC. ESI (calc. 640) *m/z* found 640.1 = [M^+].

Myr (TAMRA) (2) ($CH_3(CH_2)_{12}$COO-AC(thiopyridyl)K(TAMRA) was obtained by mixing 1 eq (20 mg) of ($CH_3(CH_2)_{12}$COO-AC(thiopyridyl)K with 5 eq of 5(6)-carboxytetramethylrhodamine (TAMRA), 5 eq of HBTU and 10 eq of N,N-Diisopropylethylamine in 10 ml DMF at RT for 3 hours. The reaction was monitored and the compound purified by RP-HPLC. ESI (calc.1052) *m/z* found: 526.3= [M^{2+}]; 1052.0 = [M^+].

All cleavable compounds Myr-s-s-Life (5), pHLIP-s-s-Life (6) and TAT-s-s-Life (7) (see scheme in Figure 1) were obtained by mixing 1 eq of each carrier (Myr(TAMRA) (2), pHLIP(TAMRA) (3) and TAT(TAMRA) (4)) with 2 eq of Lifeact(FAM) (1) in DMF (0.5 mL

each mg of Lifeact(FAM)) at RT for 4 hours. All reactions were monitored by analytical RP-HPLC, the compounds were purified by semipreparative RP-HPLC and identified by mass spectrometry. ESI Myr-s-s-Life (5) (calc 3494) *m/z* found 700.0=[M^{5+}]; 874.7=[M^{4+}]; 1166.0=[M^{3+}]; 3494.0=[M^{+}]. pHLIP-s-s-Life (6) (calc 7318) *m/z* found 1220.6=[M^{6+}]; 1464.6=[M^{5+}]; 1830.7=[M^{4+}]; 7318.0 [M^{+}]. TAT-s-s-Life (7) (calc 4856) found *m/z* 608.0=[M^{8+}]; 694.8=[M^{7+}]; 810.5=[M]$^{6+}$; 972.4=[M^{5+}].

Probes were diluted in 150 μM solutions in Tyrode's buffer (134.0 mM NaCl, 2.90 mM KCl, 0.34 mM Na_2HPO_4:12H_2O, 12.0 mM $NaHCO_3$, 20.0 mM Hepes, 1.0 mM $MgCl_2$, pH 7.4). Concentrations were checked by comparing UV-Vis bands of the dyes and aliquots were kept at -20°C until their use for cell biology studies.

Buffer and Solutions

Following buffers were employed for cell biology experiments: ACD (acid-citratedextrose 10% v/v, 85 mM sodium citrate, 110 mM glucose, 80 mM citric acid,); Tyrode's buffer 7.4 (134.0 mM NaCl, 2.90 mM KCl, 0.34 mM Na_2HPO_4:12H_2O, 12.0 mM $NaHCO_3$, 20.0 mM Hepes, 1.0 mM $MgCl_2$, pH 7.4); Tyrode's buffer 6.5 (same than Tyrode buffer but pH adjusted to 6.5); modified Tyrode's buffer 7.4 (3 mL of ACD added to 25 mL of Tyrode's buffer 7.4); Tyrode's buffer 7.4+G (5 mM Glucose in Tyrode's buffer 7.4); Tyrode's buffer 6.5+G (5 mM Glucose in Tyrode's buffer 6.5).

Preparation of human platelets

All buffer and solution were pre-warmed at 37°C. 10 mL of fresh human blood was obtained into sodium citrate (10% v/v) from healthy volunteer by venipuncture. 1mL of ACD was added to the blood and the resulting suspension was centrifuged for 20 minutes at room temperature in polypropylene tubes (200 × g). The yellow supernatant (platelet rich plasma - PRP) was transferred in a falcon tube, 5 μL of prostacyclin (PGI2, 1 mg/mL) were added and the suspension was centrifuged for 10 minutes at room temperature (1000 × g). The resulting supernatant was removed, the pellet was re-suspended in 15 mL of modified

Tyrode's buffer 7.4 and the suspension was divided in two aliquots. PGI2 (3 µl) was added to each suspension followed by centrifugation for 10 minutes at room temperature (1000 x g). The resultant supernatants were removed and the two pellets were separately re-suspended in Tyrode's buffer 7.4+G and Tyrode's buffer 6.5+G (for following incubation with pHLIP-Lifeact systems only). Cells were counted and diluted to 5×10^8 cell/mL and allowed to rest for 30 min.

Preparation of mouse megakaryocytes

Recombinant murine stem cell factor and thrombopoietin were purchased from PeproTech. Sheep anti–rat IgG Dynabeads, biotin-conjugated rat anti–mouse CD45R/B220, purified rat anti–mouse CD16/CD32 antibodies were from BD Pharmingen. Anti-mouse Ly-6G and biotin anti–mouse CD11b antibodies were from eBioscience. StemPro, DMEM medium and sheep anti–rat IgG Dynabeads were from Life Technologies.

Mature megakaryocytes (MKs) from mouse bone marrow (BM) were defined as the population of cells generated using the methodology described previously (Dumon et al., 2006; Lecine et al., 1998; Mazharian et al., 2010). In brief, BM cells were obtained from femurs and tibias of mice by flushing with DMEM medium, and cells expressing one or more of the lineage-specific markers on their surface ($CD16/CD32^+$, $Gr1^+$, $B220^+$, or $CD11b^+$) were depleted using immunomagnetic beads (sheep anti–rat IgG Dynabeads). The remaining population was cultured in 2.6% serum-supplemented StemPro medium with 2 mM L-glutamine, penicillin/streptomycin, and 20 ng/mL of murine stem cell factor at 37°C under 5% CO_2 for 2 days. Cells were then cultured for a further 4 days in the presence of 20 ng/mL of stem cell factor and 50 ng/mL of thrombopoietin. After 4 days of culture in the presence of thrombopoietin, the cell population was enriched in mature MKs using a 1.5%/3% BSA gradient under gravity (1*g*) for 45 minutes at room temperature, as described previously (Mazharian et al., 2010).

Spreading on fibrinogen and slides preparation

Platelet suspensions were further diluted to 2x10^7 cell/mL and divided in 300 μL aliquots in different eppendorf. Different volumes of 150 μM stock solutions of probes were added to each aliquot to achieve final 0.5-20 μM concentration range of probe. Tyrode's buffer pH 6.5+G was employed for pHLIP-Lifeact probes and Tyrode's buffer pH 7.4+G was employed for other probes. Cells were incubated with the compounds for 30 min at 37°C, suspensions were transferred on glass coverslips pre-coated with fibrinogen (100 mg/mL in PBS at 4°C overnight) and cell spreading was allowed for 45 min at 37°C. Cells were washed with PBS, fixed with 10% formalin for 10 minutes, treated with NH_4Cl_2 (50 mM), to quench residual formalin, and washed again with PBS and deionized water. Coverslips were mounted on glass slides using Hydromount and stored at 4 °C until imaged. Exactly the same procedure was employed to stain megakaryocytes except that after prior fixation and mounting incubation for 30 min at at 37°C, cells were spin down for 5 min at 1200 rpm, re-suspended in medium and spreading was allowed for 3 hours.

For control with phalloidin, platelet suspension (2x10^7 cell/mL in Tyrode's buffer 7.4+G) was allowed to spread on glass coverslips, pre-coated with fibrinogen, for 45 min at 37°C, fixed with formalin, permealized with 0.1% Triton X-100 in PBS and stained with 15 nM Alexa Fluor® 488 Phalloidin for 30 min at room temperature. Stained cells were washed with PBS and deionized water and mounted on slides for microscopy.

Attempts of live imaging were performed by allowing spreading of platelets in chambered slides for live cell microscopy. Washes to remove the excess of probe were attempted in two ways: i) by directly washing spread cells on fibrinogen or ii) after the 30 min incubation by precipitating with PGI2 and washing the pellets prior spreading .

Confocal reflection and fluorescence microscopy.

To allow proper comparison between different compounds, same microscope parameters (PMT, enlargement, laser power) were employed in all the experiments (at least differently indicated). Samples were imaged using the 488 nm laser line (Ar/ArKr laser), 543 nm and

633 nm laser line (He/Ne laser) on a Leica DMIRE 2 laser scanning confocal microscope with 63x, 1.4 N/A. oil objective. Reflected light was collected at 478 – 498 nm excitation of 488 nm. Fluorescence emission was collected at 510 - 540 nm for FAM and 565 - 585 nm for TAMRA. Images were acquired using the Leica TSC SP2software. Post-imaging analyses (cell selections, mean area and mean fluorescence intensities) were performed using ImageJ 1.48.

Supplemental References

Dumon, S., Heath, V.L., Tomlinson, M.G., Gottgens, B., and Frampton, J. (2006). Differentiation of murine committed megakaryocytic progenitors isolated by a novel strategy reveals the complexity of GATA and Ets factor involvement in megakaryocytopoiesis and an unexpected potential role for GATA-6. Exp Hematol *34*, 654-663.

Lecine, P., Blank, V., and Shivdasani, R. (1998). Characterization of the hematopoietic transcription factor NF-E2 in primary murine megakaryocytes. J Biol Chem *273*, 7572-7578.

Mazharian, A., Thomas, S.G., Dhanjal, T.S., Buckley, C.D., and Watson, S.P. (2010). Critical role of Src-Syk-PLC gamma 2 signaling in megakaryocyte migration and thrombopoiesis. Blood *116*, 793-800.
